# The Prevalence and Associated Factors of Compassion Satisfaction and Compassion Fatigue Among Clinical Nurses in China

**DOI:** 10.1155/jonm/8097321

**Published:** 2025-12-18

**Authors:** Tian Xiong, Jing Wang, Mei Wang

**Affiliations:** ^1^ Department of Nursing, Tongji Hospital, Tongji Medical College, Huazhong University of Science and Technology, Wuhan, Hubei, China, hust.edu.cn

**Keywords:** burnout, compassion fatigue, compassion satisfaction, secondary traumatic stress

## Abstract

**Background:**

Compassion satisfaction and compassion fatigue are critical to nurses’ well‐being and patient care. In China, factors such as high workloads and healthcare reforms may elevate nurses’ risk for compassion fatigue.

**Aims:**

To assess the prevalence and influencing factors of compassion satisfaction and compassion fatigue among Chinese clinical nurses.

**Methods:**

In this cross‐sectional study, 581 registered nurses completed surveys on demographics, work‐related factors, Pittsburgh Sleep Quality Index, Psychology Capital Questionnaire, Perceived Social Support Scale, and Professional Quality of Life Scale. Data were analyzed using descriptive statistics, nonparametric tests, and multiple linear regression.

**Results:**

The prevalence of low, average, and high levels was 5.7%, 79.2%, and 15.1% for compassion satisfaction; 31.0%, 67.6%, and 1.4% for burnout; and 34.8%, 63.5%, and 1.7% for secondary traumatic stress, respectively. Multiple linear regression analysis revealed that compassion satisfaction (*R* ^2^ = 54.3%), sleep quality (*β* = −0.09, *p* = 0.005), psychological capital (*β* = 0.540, *p* < 0.001), and social support (*β* = 0.215, *p* < 0.001) were significant predictors. Similarly, burnout (*R* ^2^ = 46.9%), sleep quality (*β* = 0.224, *p* < 0.001), psychological capital (*β* = −0.329, *p* < 0.001), and social support (*β* = −0.289, *p* < 0.001) were significant predictors. Secondary traumatic stress (*R* ^2^ = 8.0%), sleep quality (*β* = 0.231, *p* < 0.001), and social support (*β* = −0.13, *p* = 0.009) were also significant predictors.

**Conclusions:**

Chinese clinical nurses experience significant compassion fatigue. Sleep quality, psychological capital, and social support are key factors associated with their professional quality of life, highlighting targets for intervention by nursing management.

**Implications for Nursing Management:**

It is important for nursing managers to recognize the susceptibility of nurses to compassion fatigue and develop specific strategies to alleviate burnout and secondary traumatic stress while fostering compassion satisfaction.

## 1. Introduction

As a direct provider for medical services, nurses play a pivotal role in improving patient’s health and preventing nurse–patient conflicts. Unlike other medical staff members, nurses are more susceptible to compassion fatigue [1, 2]. It is worthy of note that physical demands, extended work hours, irregular schedules, and frequent exposure to suffering and death can significantly impact nurses’ well‐being [3, 4]. The incidence of compassion fatigue among clinical nurses reaches up to 75.9% or higher [5–8]. In recent years, compassion fatigue among nurses has received considerable attention because of the growing evidence that it can adversely affect various aspects of nurses, reducing nursing performance outcomes, job satisfaction, and increasing turnover rates [3, 4, 9, 10]. Compassion fatigue is critical for ensuring the health and safety of nurses, which is coupled with the enhanced quality of patient care, and improved outcomes within health department administration [11, 12].

Compassion fatigue is commonly measured using the Professional Quality of Life Scale (Pro‐QOL) developed by Stamm [13], which assesses three constructs: compassion satisfaction, burnout, and secondary traumatic stress. Compassion satisfaction represents a positive dimension of professional quality of life, denoting pleasure and fulfillment from assisting others [14]. It encompasses emotional accomplishment through effective care provision, colleague support, and workplace contribution [15]. Higher compassion satisfaction enhances role fulfillment and work engagement [4], mitigates adverse effects of burnout and secondary traumatic stress [13], and negatively correlates with nurse turnover [4, 10].

Conversely, compassion fatigue constitutes a negative dimension, characterized by the exhaustion and dysfunction resulting from prolonged emotional stress exposure [16]. This construct comprises two components: (1) burnout, defined as physical and emotional depletion from value–action misalignment [17], and (2) secondary traumatic stress, characterized by the trauma symptoms mirroring patients’ experiences (e.g., intrusive thoughts, avoidance behaviors, and hyperarousal) [18]. Collectively, these reactions impair nurses’ care capacity and professional quality of life [3, 9].

Based on the Pro‐QOL model and existing literature, we hypothesize that work‐related factors, personal characteristics, and psychosocial factors collectively influence professional quality of life. Work‐related factors (such as work environment, department, and working schedules) are significantly associated with nurses’ professional quality of life [3, 4, 7, 10]. Positive work environment reduces compassion fatigue and enhances compassion satisfaction [10, 12]. In particular, emergency and oncology departments consistently demonstrate elevated stress levels among nursing staff [11, 19]. Workloads exceeding 40 weekly hours or more than 8 monthly night shifts elevate compassion fatigue risk [7]. Such chronic exposure sustains physiological arousal, hinders emotional processing, and disrupts self‐care, thereby accelerating emotional exhaustion and diminishing professional accomplishment.

Personal variables including age, seniority, experience, education, professional title, sleep quality, and exercise frequency influence nurses’ professional quality of life with varying patterns [4, 7, 11, 20]. Nurses with less than 10 years of experience demonstrated lower compassion satisfaction, while more experienced nurses tended to exhibit higher compassion fatigue, which may stem from greater involvement in complex procedures and heightened caregiving responsibilities [14]. Furthermore, junior nurses are more susceptible to performance pressure and burnout [11]. In contrast, senior nurses show divergent outcomes across settings, such as higher compassion satisfaction in emergency departments [11], but greater secondary traumatic stress in hospice care [11], yet experiencing increased secondary traumatic stress in emotionally demanding areas such as hospice care [7]. Sleep hygiene and regular physical activity act as protective factors against compassion fatigue by enhancing resilience, wherein restorative sleep mediates this buffering effect [4]. Inadequate sleep correlates with chronic fatigue and reduced job performance, impairing both work quality and responsiveness to patients [21]. Sufficient rest, by contrast, enhances the ability to gain fulfillment from compassionate care [21]. As an established indicator of mental health, sleep quality significantly influences the development and persistence of compassion fatigue [22].

Psychological capital plays a critical role in determining professional quality of life. It is characterized as a positive psychological state of individuals and consists of four major components: hope, self‐efficacy, resilience, and optimism [23]. Hope reflects a positive outlook and determination toward achieving one’s goals; self‐efficacy denotes the belief in one’s capacity to successfully execute tasks and accomplish desired outcomes; resilience refers to the ability to adapt positively in the face of adversity and recover from difficulties; optimism, meanwhile, represents a cognitive tendency to expect favorable outcomes and to interpret life events in a positive manner [23]. Nurses possessing elevated levels of psychological capital tend to maintain a positive outlook toward the future and demonstrate considerable resilience in the face of stress [24]. Rather than being overwhelmed by challenges, they often perceive them as sources of motivation [5]. Such nurses exhibit stronger organizational commitment, are less likely to leave their positions, and consistently strive to meet high performance expectations in their professional roles [3]. Psychological capital mitigates the effects of job demands and fosters intrinsic motivation, which subsequently promotes compassion satisfaction while reducing compassion fatigue [25].

Multidimensional social support refers to assistance from family, friends, supervisors, and colleagues [26]. It can help people enhance individuals’ problem‐solving abilities, facilitate adaptation to stress, and mitigate the adverse effects on physical and mental health [2]. Nurses who perceive stronger support from family and friends are better able to concentrate fully at work, release negative emotions appropriately, and confront challenges with greater confidence, thereby reducing feelings of exhaustion and detachment [8, 27]. Besides, support from supervisors and co‐workers serves as a valuable job resource that alleviates emotional exhaustion and enhances a sense of personal meaningfulness in nursing practice [11, 14]. Thus, social support functions as a protective factor, associated with higher compassion satisfaction and lower compassion fatigue among clinic nurses [2].

Clinic nurses in China might be especially susceptible to compassion fatigue, influenced not just by their demanding workloads but also by the pressure for high service quality, conflicts between hospitals and patients, and continuous reforms in healthcare [4]. To address this issue, our study describes the current status of professional quality of life among nurses in China. We aimed to (1) investigate the prevalence of compassion satisfaction and compassion fatigue and (2) identify the factors influencing them, thereby informing potential support strategies.

## 2. Methods

### 2.1. Ethical Consideration, Study Design, and Participants

Following institutional review board approval, written informed consent was obtained from all participants. We conducted an exploratory cross‐sectional study employing convenience sampling at a university‐affiliated tertiary hospital in Wuhan. This facility serves as one of China’s National Major Public Health Events Medical Centers, the principal healthcare institution in central China. Participants could voluntarily participate in this study and could withdraw at any time if they wanted. The inclusion criteria were as follows: (1) registered nurses, (2) at least 1 year of clinic nursing experience (this criterion was set to target nurses who may have completed the initial transition period and whose professional quality of life and psychological adaptation were considered more stable and reflective of the established clinical workforce [10]), and (3) consent to participate in the study. Exclusion criteria included the following: (1) absence from clinical nursing work in the past 6 months and (2) individuals who were unwilling to participate in this study. The sample size for multiple regression analysis with 20 independent variables was calculated via G∗Power (Version 3.1). Based on an effect size of 0.15, alpha of 0.05, and power of 0.8, the initial estimate was 157 participants. Accounting for a 20% anticipated response loss, the adjusted sample size required was 197. To ensure robust data collection and address potential covariates, we ultimately recruited 581 participants.

### 2.2. Data Collection

Data were collected from May to June 2024 by the researchers. Initially, directors and head nurses of the university‐affiliated hospital were informed of the purpose of the research. The researchers then presented the questionnaires and provided clear, standardized instructions on how to complete them, including explanations of key terms and response scales. Prior to the main study, a pilot test was conducted with 21 nurses to assess clarity, comprehension, and time burden (approx. 13 min), leading to minor refinements. Next, the researchers distributed a Wenjuanxing link, an online platform in China, which directed participants to the electronic research questionnaires. Finally, the participants were guided to complete the questionnaires step by step. After submission, the researchers downloaded the data through the online platform for analysis. The downloaded data underwent an initial completeness and consistency check. It is important to note that Wenjuanxing is a secure platform, minimizing the risk of data loss.

### 2.3. Variables and Instruments

#### 2.3.1. General Questionnaire

A general questionnaire was developed to collect the demographic characteristics and work‐related factors of the participants, including gender, age, education level, years of nursing work, employment type, professional title, shift work, exercise frequency, marital status, living status, self‐rated health, and alcohol and tobacco use.

#### 2.3.2. Pittsburgh Sleep Quality Index (PSQI)

The PSQI comprises 7 subscales: subjective sleep quality, sleep latency, sleep duration, sleep efficiency, sleep disturbances, use of sleep medications, and daytime drowsiness [28]. Each subscale is scored from 0 (*no difficulty*) to 3 (*severe difficulty*), with the total score ranging from 0 to 21. A score greater than five indicates significant sleep disturbance. The PSQI has been extensively validated and widely utilized in Chinese nurses [29, 30]. The Chinese version of the PSQI was used in this study. The Cronbach’s *α* for the total scale in our sample was 0.774.

#### 2.3.3. Psychology Capital Questionnaire (PCQ‐24)

The PCQ‐24 evaluates the employees’ perceptions of positive psychological capital [23]. It consists of 24 items scored on a 6‐point scale ranging from 1 (*strongly disagree*) to 6 (*strongly agree*). It includes four subscales as follows: hope, self‐efficacy, resilience, and optimism. High scores indicate high psychological capital, which means the individuals are more hopeful, self‐efficacious, psychologically more resilient, and more optimistic. The PCQ‐24 has been extensively employed among nurses in China [31]. We employed the validated Chinese version of the PCQ‐24. In this study, the Cronbach’s *α* was 0.851 for the overall scale. The values for the subscales were as follows: hope (0.838), self‐efficacy (0.835), resilience (0.776), and optimism (0.766).

#### 2.3.4. Perceived Social Support Scale (PSSS)

The PSSS was used to evaluate an individual’s experience of support from families, friends, and other sources [26]. The scale comprises 12 items. Each item is scored on a scale from 1 (*strongly disagree*) to 7 (*strongly agree*), with a total score ranging from 12 to 84. A higher score indicates a greater level of perceived social support. The scale was administered in its standardized Chinese form [32]. In this study, the overall Cronbach’s *α* was 0.908. The subscale alphas were family support (0.891), friend support (0.876), and other support (0.865).

#### 2.3.5. Pro‐QOL

The Pro‐QOL was used to assess compassion satisfaction, burnout, and secondary traumatic stress of nurses [13]. We used the Chinese version of the Pro‐QOL [33]. This scale comprises three subscales, each employing a five‐point Likert scale ranging from 1 (*never*) to 5 (*very often*) [33]. A higher score on the compassion satisfaction subscale indicates greater satisfaction related to a nurse’s effectiveness; a higher score on the burnout subscale signifies an increased risk of burnout. In addition, a higher score on the secondary traumatic stress subscale suggests that nurses may need to reflect on their feelings regarding their job and work environment [33]. For each subscale, the scoring ranges were defined as follows: low (scores of 22 or less), average (scores ranging from 23 to 41), and high (scores of 42 and above) in relation to compassion satisfaction, burnout, and secondary traumatic stress [33]. The scale demonstrates acceptable construct validity and has been widely employed among nurses in China [4, 7, 33]. In this study, the Cronbach’s *α* values for the subscales of compassion satisfaction, burnout, and secondary traumatic stress were 0.821, 0.733, and 0.717, respectively.

### 2.4. Statistical Analysis

SPSS (Version 18.0, IBM) was employed. Categorical variables were described by frequency and composition ratio *n* (%). The distribution of continuous variables was checked for normality using the Kolmogorov–Smirnov test. As they had non‐normal distributions, the median (25th–75th percentile) (Md [P_25_–P_75_]) was used to describe the continuous variables using the Mann–Whitney *U* test and the Kruskal–Wallis *H* test for univariate analysis, and the Spearman’s correlation analysis between bivariate variables. Multiple linear regression analysis was conducted to identify factors influencing compassion satisfaction, burnout, and secondary traumatic stress, with evaluation of multicollinearity. All tests were two‐tailed, and *p* < 0.05 was considered statistically significant.

## 3. Results

### 3.1. Demographic Variables

A total of 590 nurses were invited to participate in this study. After excluding nine nurses who declined to complete the questionnaires, 581 were included in the final analysis, yielding a valid response rate of 98.5%. The median age of the participants was 34.00 (30.00–40.00) years, with an overall range of 23–58 years. As shown in Table 1, the majority of participants were female (*n* = 570, 98.1%), aged 25–34 years (*n* = 272, 46.8%), married (*n* = 464, 79.9%), and held a bachelor’s degree (*n* = 544, 93.6%). Regarding work characteristics, a substantial proportion had worked for at least nine years (*n* = 391, 67.3%) and were employed on night shifts (*n* = 467, 80.4%). In terms of health behaviors, only a small number reported cigarette smoking (*n* = 14, 2.4%) or alcohol consumption (*n* = 65, 11.2%), while half (*n* = 296, 50.9%) did not engage in regular exercise in the past three months.

**Table 1:** Demographic and work‐related characteristics (*n* = 581).

Variables	*n*	(%)
Gender		
Male	11	1.9
Female	570	98.1
Age (years)		
< 25	24	4.1
25–34	272	46.8
35–44	216	37.2
≥ 45	69	11.9
Education level		
Below bachelor	6	1.0
Bachelor	544	93.6
Master’s or above	31	5.3
Form of employment		
Staff	94	16.2
Contract	458	78.8
Standardized training	29	5.0
Professional title		
Primary	256	44.1
Intermediate	309	53.2
Senior	16	2.8
Working position		
Head nurse	36	6.2
General nurse	545	93.8
Years of nursing work		
≤ 4	57	9.8
5–8	133	22.9
9–14	202	34.8
≥ 15	189	32.5
Night shift work		
No	114	19.6
Yes	467	80.4
Length of commute, h		
< 0.5	123	21.2
0.5–1	211	36.3
1–1.5	133	22.9
> 1.5	114	19.6
Marital status		
Unmarried	107	18.4
Married	464	79.9
Divorced/widowed	10	1.7
Children, num.		
0	149	25.6
1	321	55.2
2	111	19.1
Living status		
Live alone	95	16.4
Live with family	486	83.6
Exercise frequency		
Never	296	50.9
Rarely (≥ 1/quarter)	157	27.0
Occasionally (≥ 1/month)	51	8.8
Frequently (≥ 1/week)	77	13.3
Smoking		
No	567	97.6
Yes	14	2.4
Alcohol		
No	516	88.8
Yes	65	11.2
Chronic diseases		
No	89	15.3
Yes	492	84.7
Self‐rated health		
Good	341	58.7
Fair	212	36.5
Poor	28	4.8

### 3.2. Descriptive Analysis of the Four Scales

The prevalence of low, average, and high levels of compassion satisfaction among participants was 5.7% (*n* = 33), 79.2% (*n* = 460), and 15.1% (*n* = 88), respectively. For burnout, 31.0% (*n* = 180) of nurses were at low risk, 67.6% (*n* = 393) at average risk, and 1.4% (*n* = 8) at high risk. Similarly, the prevalence of secondary traumatic stress was 34.8% (*n* = 202) for low, 63.5% (*n* = 369) for average, and 1.7% (*n* = 10) for high levels. The median scores were 32.00 (P_25_–P_75_: 29.50–39.00) for compassion satisfaction, 26.00 (P_25_–P_75_: 21.00–30.00) for burnout, and 24.00 (P_25_–P_75_: 21.00–29.00) for secondary traumatic stress. The median PSQI score was 6.00 (P_25_–P_75_: 4.00–8.00). The prevalence of sleep disorders among participants was 52.15% (*n* = 303). The median PCQ score was 4.42 (P_25_–P_75_: 4.00–4.96). The median PSSS score was 63.00 (P_25_–P_75_: 48.00–72.00) (Table 2).

**Table 2:** Median and quartile of professional quality of life, sleep quality, psychological capital, and perceived social support.

Variables	Median	P_25_–P_75_
(1) Compassion satisfaction	32.00	29.50–39.00
(2) Burnout	26.00	21.00–30.00
(3) Secondary traumatic stress	24.00	21.00–29.00
(4) Sleep quality	6.00	4.00–8.00
(5) Psychological capital	4.42	4.00–4.96
(5.1) Hope	4.50	4.00–5.00
(5.2) Self‐efficacy	4.67	4.00–5.00
(5.3) Resilience	4.17	4.00–4.67
(5.4) Optimism	4.17	4.00–4.67
(6) Perceived social support	63.00	48.00–72.00
(6.1) Family support	22.00	16.00–24.00
(6.2) Friend’s support	21.00	16.00–24.00
(6.3) Other support	20.00	16.00–24.00

*Note:* P_25_–P_75_, 25th–75th percentile.

### 3.3. Univariate Analysis of the Factors Associated With Compassion Satisfaction, Burnout, and Secondary Traumatic Stress

The results of the Mann–Whitney *U* test and Kruskal–Wallis *H* test indicated that nurses who were ≥ 45 years old (*H* = 12.808, *p* = 0.005), married (*H* = 18.217, *p* < 0.001), staff (*H* = 13.389, *p* = 0.001), senior (*H* = 17.258, *p* < 0.001), head nurses (*Z* = −3.573, *p* < 0.001), had ≥ 15 years of nursing experience (*H* = 13.644, *p* = 0.003), had two children (*H* = 15.794, *p* < 0.001), lived with family (*Z* = −3.208, *p* = 0.001), frequently exercise (*H* = 8.607, *p* = 0.035), had no chronic diseases (*Z* = −2.329, *p* = 0.020), and had good self‐rated health (*H* = 42.861, *p* < 0.001) were associated with higher levels of compassion satisfaction (Table 3).

**Table 3:** Univariate analyses of the factors associated with compassion satisfaction, burnout, and secondary traumatic stress.

Variables	Compassion satisfaction Md (P_25_–P_75_)	Burnout Md (P_25_–P_75_)	Secondary traumatic stress Md (P_25_–P_75_)
Gender			
Male	34.00 (30.00–41.00)	25.00 (22.00–30.00)	23.00 (21.00–32.00)
Female	32.00 (29.00–39.00)	26.00 (21.00–30.00)	24.00 (21.00–29.00)
*Z*	−0.235	−0.004	−0.377
*p*	0.814	0.997	0.706
Age (years)			
< 25	31.00 (26.05–35.75)	27.50 (22.25–30.75)	23.00 (20.25–28.25)
25–34	32.00 (29.00–38.75)	26.00 (21.00–30.00)	24.00 (21.00–30.00)
35–44	32.00 (29.00–38.75)	27.00 (22.00–30.00)	25.00 (21.00–29.00)
≥ 45	37.00 (30.00–41.00)	23.00 (20.00–28.00)	24.00 (21.00–29.00)
*H*	12.808	11.022	1.250
*p*	0.005^∗^	0.012^∗^	0.741
Education level			
Below bachelor	33.00 (26.25–35.25)	23.00 (20.75–30.50)	20.00 (16.50–30.75)
Bachelor	32.00 (29.00–39.00)	26.00 (21.00–30.00)	24.00 (21.00–29.00)
Master’s or above	33.00 (30.00–38.00)	26.00 (22.00–30.00)	27.00 (22.00–30.00)
*H*	0.887	0.160	3.675
*p*	0.642	0.923	0.159
Form of employment			
Staff	34.00 (30.00–40.25)	24.00 (20.00–29.00)	24.00 (21.00–28.00)
Contract	32.00 (29.00–39.00)	26.00 (21.00–30.00)	24.00 (21.00–29.00)
Standardized training	30.00 (26.00–34.50)	28.00 (23.50–32.00)	23.00 (21.00–29.50)
*H*	13.389	9.538	0.370
*p*	0.001^∗^	0.008^∗^	0.831
Professional title			
Primary	31.00 (29.00–38.00)	26.00 (22.00–30.00)	24.00 (21.00–29.00)
Intermediate	33.00 (30.00–39.50)	25.00 (21.00–29.00)	25.00 (21.00–29.00)
Senior	39.50 (34.00–45.00)	20.00 (16.25–22.75)	24.50 (23.25–27.00)
*H*	17.258	13.619	0.295
*p*	< 0.001^∗∗^	0.001^∗^	0.863
Working position			
Head nurse	38.00 (33.00–41.00)	22.50 (20.00–27.50)	24.00 (22.00–27.75)
General nurse	32.00 (29.00–39.00)	26.00 (21.00–30.00)	24.00 (21.00–29.00)
*Z*	−3.573	−2.514	−0.110
*p*	< 0.001^∗∗^	0.012^∗^	0.912
Years of nursing work			
≤ 4	31.00 (25.00–35.00)	28.00 (23.00–30.50)	24.00 (21.00–30.00)
5–8	31.00 (29.00–38.00)	26.00 (22.00–30.00)	25.00 (21.00–30.00)
9–14	33.00 (30.00–39.25)	25.00 (20.00–29.00)	24.00 (20.00–29.00)
≥ 15	33.00 (30.00–40.00)	25.00 (20.00–30.00)	24.00 (21.00–29.00)
*H*	13.644	9.703	1.608
*p*	0.003^∗^	0.021^∗^	0.658
Night shift work			
No	32.00 (29.00–39.00)	26.00 (21.00–29.00)	24.00 (21.00–29.00)
Yes	33.50 (30.00–40.00)	24.00 (20.00–30.00)	25.00 (21.00–30.00)
*Z*	−1.607	−1.048	−0.778
*p*	0.108	0.295	0.436
Length of commute, h			
< 0.5	32.00 (29.00–37.00)	26.00 (22.00–30.00)	25.00 (21.00–30.00)
0.5–1	32.00 (29.00–39.00)	26.00 (21.00–30.00)	24.00 (21.00–28.00)
1–1.5	32.00 (30.00–39.00)	26.00 (21.50–29.00)	26.00 (21.50–29.50)
> 1.5	33.00 (30.00–39.25)	25.00 (20.00–29.00)	24.00 (20.00–29.00)
*H*	2.828	1.757	6.678
*p*	0.419	0.624	0.083
Marital status			
Unmarried	30.00 (26.00–35.00)	28.00 (23.00–30.00)	25.00 (21.00–30.00)
Married	35.00 (30.00–46.00)	22.50 (18.50–30.00)	24.00 (21.00–29.00)
Divorced/widowed	33.00 (30.00–39.00)	25.00 (21.00–29.00)	26.00 (18.75–30.00)
*H*	18.217	12.141	1.161
*p*	< 0.001^∗∗^	0.002^∗^	0.560
Children, num.			
0	31.00 (27.50–35.00)	27.00 (23.00–30.00)	25.00 (21.00–30.00)
1	32.00 (30.00–39.00)	25.00 (21.00–29.00)	24.00 (21.00–29.00)
2	34.00 (30.00–40.00)	25.00 (20.00–29.00)	24.00 (21.00–29.00)
*H*	15.794	10.471	1.610
*p*	< 0.001^∗∗^	0.005^∗^	0.447
Living status			
Live alone	30.00 (26.00–35.00)	27.00 (22.00–30.00)	25.00 (21.00–30.00)
Live with family	33.00 (30.00–39.00)	25.00 (21.00–29.25)	24.00 (21.00–29.00)
*Z*	−3.208	−2.469	−1.175
*p*	0.001^∗^	0.014^∗^	0.240
Exercise frequency			
Never	31.00 (29.00–38.00)	26.00 (22.00–30.00)	24.00 (21.00–29.00)
Rarely	33.00 (30.00–38.50)	25.00 (22.00–29.00)	25.00 (22.00–30.00)
Occasionally	34.00 (29.00–40.00)	23.00 (21.00–29.00)	21.00 (18.00–28.00)
Frequently	35.00 (30.00–40.00)	23.00 (20.00–28.00)	24.00 (21.00–28.00)
*H*	8.607	10.277	7.150
*p*	0.035^∗^	0.016^∗^	0.067
Smoking			
No	32.00 (29.00–39.00)	26.00 (21.00–30.00)	24.00 (21.00–29.00)
Yes	35.00 (29.75–47.00)	25.00 (18.50–30.00)	24.50 (22.50–30.00)
*Z*	−1.043	−0.100	−0.408
*p*	0.297	0.920	0.683
Alcohol			
No	32.00 (29.25–39.00)	26.00 (21.00–30.00)	24.00 (21.00–29.00)
Yes	34.00 (29.50–40.00)	25.00 (20.50–30.00)	24.00 (20.50–29.00)
*Z*	−0.638	−0.175	−0.031
*p*	0.523	0.861	0.975
Chronic diseases			
No	32.00 (30.00–39.00)	25.00 (21.00–29.00)	24.00 (21.00–29.00)
Yes	30.00 (28.00–38.00)	28.00 (23.00–30.00)	22.00 (22.00–29.00)
*Z*	−2.329	−3.327	−1.812
*p*	0.020^∗^	0.001^∗^	0.070
Self‐rated health			
Good	34.00 (30.00–40.00)	24.00 (20.00–28.00)	23.00 (20.00–29.00)
Fair	30.00 (28.00–36.00)	27.00 (23.00–30.00)	25.00 (21.00–29.00)
Poor	30.50 (29.00–33.00)	30.00 (28.25–32.00)	28.50 (25.00–30.00)
*H*	42.861	60.314	16.293
*p*	< 0.001^∗∗^	< 0.001^∗∗^	< 0.001^∗∗^

*Note:* Md (P_25_–P_75_), median (25th–75th percentile).

^∗^ *p* < 0.05.

^∗∗^ *p* < 0.001.

Similarly, these nurses who were ≥ 45 years old (*H* = 11.022, *p* = 0.012), married (*H* = 12.141, *p* = 0.002), staff (*H* = 9.538, *p* = 0.008), senior (*H* = 13.619, *p* = 0.001), head nurses (*Z* = −2.514, *p* = 0.012), had 9–14 years of clinical nursing experience (*H* = 9.703, *p* = 0.021), had two children (*H* = 10.471, *p* = 0.005), lived with family (*Z* = −2.469, *p* = 0.014), frequently exercise (*H* = 10.277, *p* = 0.016), had no chronic diseases (*Z* = −3.327, *p* = 0.001), and had good self‐rated health (*H* = 60.314, *p* < 0.001) also demonstrated lower levels of burnout (Table 3).

Regarding secondary traumatic stress, nurses with good self‐rated health (*H* = 16.293, *p* < 0.001) had lower levels of secondary traumatic stress (Table 3).

### 3.4. Spearman’s Correlation Analysis of Professional Quality of Life, PSQI, PCQ, and PSSS

Spearman’s correlation analyses showed that the PSQI was negatively correlated with compassion satisfaction (*r* = −0.292, *p* < 0.001). However, it was positively correlated with burnout (*r* = 0.402, *p* < 0.001) and secondary traumatic stress (*r* = 0.274, *p* < 0.001). On the contrary, the PSSS was positively correlated with compassion satisfaction (*r* = 0.550, *p* < 0.001) and negatively correlated with burnout (*r* = −0.562, *p* < 0.001) and secondary traumatic stress (*r* = −0.233, *p* < 0.001). Lastly, the PCQ manifested positive correlations with compassion satisfaction (*r* = 0.686, *p* < 0.001) and negative correlations with burnout (*r* = −0.589, *p* < 0.001) and secondary traumatic stress (*r* = −0.172, *p* < 0.001) (Table 4).

**Table 4:** Spearman’s correlation analysis of professional quality of life, sleep quality, psychological capital, and perceived social support.

Variables	1	2	3	4	5	6
(1) Compassion satisfaction	—					
(2) Burnout	−0.774^∗∗^	—				
(3) Secondary traumatic stress	−0.157^∗∗^	0.549^∗∗^	—			
(4) Sleep quality	−0.292^∗∗^	0.402^∗∗^	0.274^∗∗^	—		
(5) Psychological capital	0.686^∗∗^	−0.589^∗∗^	−0.172^∗∗^	−0.239^∗∗^	—	
(6) Perceived social support	0.550^∗∗^	−0.562^∗∗^	−0.233^∗∗^	−0.257^∗∗^	0.568^∗∗^	—

^∗∗^ *p* < 0.001.

### 3.5. Multiple Linear Regression Model of Factors Associated With Compassion Satisfaction, Burnout, and Secondary Traumatic Stress

The results of multiple linear regression analysis are presented in Table 5. For compassion satisfaction, the model identified sleep quality (*β* = −0.09, *p* = 0.005), psychological capital (*β* = 0.540, *p* < 0.001), and perceived social support (*β* = 0.215, *p* < 0.001) as significant independent predictors, collectively explaining 54.3% of the variance.

**Table 5:** Multiple linear regression model of predictors of compassion satisfaction, burnout, and secondary traumatic stress.

Model	Variables	*b*	SE*b*	Beta	*t*	*p*	Tolerance	VIF
Compassion satisfaction^a^	Sleep quality	−0.220	0.078	−0.09	−2.832	0.005^∗∗^	0.743	1.346
	Psychological capacity	5.779	0.395	0.540	14.623	< 0.001^∗∗^	0.578	1.732
	Social support	0.121	0.021	0.215	5.814	< 0.001^∗∗^	0.575	1.740

Burnout^b^	Constant	41.742	2.498	—	16.710	< 0.001^∗∗^	—	—
	Sleep quality	0.416	0.065	0.224	6.387	< 0.001^∗∗^	0.743	1.346
	Psychological capacity	−2.734	0.331	−0.329	−8.268	< 0.001^∗∗^	0.578	1.732
	Social support	−0.126	0.017	−0.289	−7.249	< 0.001^∗∗^	0.575	1.740

Secondary traumatic stress^c^	Constant	22.370	1.902	—	11.764	< 0.001^∗∗^	—	—
	Sleep quality	0.431	0.081	0.231	5.298	< 0.001^∗∗^	0.832	1.201
	Social support	−0.058	0.022	−0.13	−2.638	0.009^∗^	0.623	1.606

*Note:* Variables included in the equations (compassion satisfaction and burnout) but with nonsignificant coefficients were age, form of employment, professional title, working position, years of nursing work, marital status, children number, living status, exercise frequency, self‐reported chronic diseases, and self‐rated health. Variables included in the equation on secondary traumatic stress but with nonsignificant coefficients were self‐rated health.

^a^ *F* = 24.008, *p* < 0.001, *R* ^2^ = 0.567, adjusted *R* ^2^ = 0.543, and Durbin–Watson value = 2.002.

^b^ *F* = 18.096, *p* < 0.001, *R* ^2^ = 0.497, adjusted *R* ^2^ = 0.469, and Durbin–Watson value = 2.075.

^c^ *F* = 11.053, *p* < 0.001, *R* ^2^ = 0.088, adjusted *R* ^2^ = 0.080, and Durbin–Watson value = 2.031.

^∗^ *p* < 0.05.

^∗∗^ *p* < 0.001.

For burnout, the model explained 46.9% of the variance, with sleep quality (*β* = 0.224, *p* < 0.001), psychological capital (*β* = −0.329, *p* < 0.001), and perceived social support (*β* = −0.289, *p* < 0.001) being significant predictors.

In the secondary traumatic stress model, which explained 8.0% of the variance, sleep quality (*β* = 0.231, *p* < 0.001) and perceived social support (*β* = −0.13, *p* = 0.009) were significant independent predictors.

## 4. Discussion

### 4.1. Current Status of Compassion Satisfaction, Burnout, and Secondary Traumatic Stress

Consistent with prior reports in China [4, 27, 33], clinical nurses in this study exhibited a professional quality of life profile characterized by moderate compassion fatigue but critically low compassion satisfaction. Compared to U.S. nurses [11, 20], Chinese nurses report significantly lower compassion satisfaction and higher compassion fatigue. Specifically, only 15.1% of nurses reported high compassion satisfaction, while the vast majority fell into the average risk category for burnout (67.6%) and secondary traumatic stress (63.5%). This pattern stands in stark contrast to U.S. nursing populations [20], where high compassion satisfaction is markedly more prevalent (44.1%), and a much larger proportion of nurses are categorized as low risk for burnout (59.4%) and secondary traumatic stress (69.4%) [20].

These pronounced disparities can be attributed to cultural, work environment, workload, and nurse characteristic variations [3, 7, 11, 27]. For instance, Chinese nurses experience a much higher prevalence of night rotating shifts (80.38% in this study vs. 28.70% in comparable US settings) [20]. These shifts cause sleep disturbance and fatigue, further exacerbated by mandatory scheduling (contrasted with US self‐scheduling practices). Compounding these issues, while exercise is linked to higher compassion satisfaction [4], 50.9% of nurses in this study reported no exercise in the past 3 months. These challenges are intensified by workplace burdens, including extended shifts [4, 7], sleep‐disrupting schedules [4, 20], and high‐acuity care [7, 11]. Collectively, these findings indicate that Chinese nurses face severe professional quality of life challenges, necessitating institutional strategies to manage compassion fatigue and enhance compassion satisfaction [27].

### 4.2. Influencing Factors of Compassion Satisfaction, Burnout, and Secondary Traumatic Stress

This study revealed that sleep quality was a significantly associated factor for compassion satisfaction, burnout, and secondary traumatic stress. PSQI scores were negatively correlated with compassion satisfaction and positively correlated with burnout and secondary traumatic stress (Table 5). The multiple linear regression model confirmed that poorer sleep quality significantly predicted lower compassion satisfaction (*β* = −0.09, *p* = 0.005), higher burnout (*β* = 0.224, *p* < 0.001), and higher secondary traumatic stress (*β* = 0.231, *p* < 0.001). As the PSQI score increases (indicating worse sleep quality), a person’s burnout and secondary traumatic stress ratings also increase, while compassion satisfaction rating decreases. Therefore, better sleep quality is associated with higher compassion satisfaction and lower burnout and secondary traumatic stress. This is consistent with the previous reports [4, 21]. Appropriate rest can enhance the ability to derive satisfaction from providing compassionate care to patients [21]. It has been well known that sleep quality is a critical indicator of mental health and has a significant impact on the development of nurses’ compassion fatigue [22]. As poor sleep quality has been related to perseveration [34], especially relevant to difficult work experiences, it is therefore quite possible that poor sleep quality contributes to the increased compassion fatigue [35]. To optimize clinic nurses’ professional quality of life and better performance at work, managers should consider physical recovery when scheduling shifts for nurses [36]. Improving sleep quality may be an important approach against compassion fatigue in future research.

We also found that psychological capacity (PCQ score) was significantly correlated with compassion satisfaction and negatively associated with compassion burnout. Regression analysis identified psychological capacity as the strongest predictor, showing a significant positive association with compassion satisfaction (*β* = 0.540, *p* < 0.001) and a significant negative association with burnout (*β* = −0.329, *p* < 0.001). This finding was consistent with a previous report [25], but psychological capacity did not emerge as a viable contributor of secondary traumatic stress in this population, as it was not retained in the final regression model for secondary traumatic stress. In a group of genetic counselors, those with low optimism exhibited increased compassion fatigue and decreased compassion satisfaction [37]. In general, nurses with a high level of positive psychological capital are unlikely to experience burnout, as positive psychological capital encompasses personal resources such as hope, optimism, resilience, and self‐efficacy [38]. A study from a tertiary hospital in Korea also demonstrated that nurses with higher positive psychological capital are unlikely experience burnout along with better nursing performance outcomes [5]. Nurses who are hopeful, optimistic, resilient, and confident, appear to achieve superior performance, as those with higher positive psychological capital are unlikely to give up or become frustrated. Instead, they are more motivated to persist in their work assignments, particularly when confronted with challenges [39]. Positive psychological capital could be a crucial buffer against the effect of burnout in hospital nurses [5]. It is feasible to achieve favorable nursing performance outcomes and reduced burnout if nurses possess adequate capacity [3]. It is essential to develop effective interventions to enhance positive psychological capital among nurses, which would enable them to manage their burnout better. Physical exercises [40], mindfulness [19], meditation, and resilience‐based training [41] were reported to be effective in mitigating nurses’ psychological distress and alleviating compassion fatigue. However, additional research would be necessary to explore meaningful strategies and programs that can be employed to foster positive psychological values.

Another key factor that can significantly influence professional quality of life is social support. This is consistent with previous studies, in which social support is positively associated with compassion satisfaction, while social support is negatively correlated to burnout and secondary traumatic stress [10, 27]. In our regression models, social support was a significant positive predictor of compassion satisfaction (*β* = 0.215, *p* < 0.001) and a significant negative predictor of both burnout (*β* = −0.289, *p* < 0.001) and secondary traumatic stress (*β* = −0.13, *p* = 0.009). Clinical nurses often encounter and empathize with patients’ traumatic experiences in their daily work. Combined with insufficient support over time, this can lead to emotional exhaustion [27]. Access to multiple forms of support from families, friends, and colleagues can strengthen their problem‐solving confidence and resilience, and this type of support would encourage them to adopt positive coping strategies, such as discussing concerns with loved ones or participating in relaxation activities to mitigate negative emotions and prevent compassion fatigue [8, 42]. Supportive nurse leaders are prone to cultivating high levels of compassion satisfaction among nurses and reducing compassion fatigue simultaneously [11, 43]. Nurse leaders must be vigilant in identifying nurses who are at higher risk for compassion fatigue and establish positive relationships with them to provide appropriate counseling and communication. These leaders play a crucial role in effectively developing strong, positive professional practice environments. By cultivating a supportive atmosphere, early recognition of compassion fatigue in nurses, along with adequate managerial support, may contribute to retaining knowledgeable, compassionate, and experienced nurses [44]. Therefore, nursing managers are highly recommended to establish an effective social support network for nurses and assist them in identifying adequate social support in their surroundings once they experience work stress and encourage clinical nurses to adopt positive coping strategies to alleviate compassion fatigue.

## 5. Limitations

This study has several limitations. First, the cross‐sectional design limits the ability to assess causal relationships between the variables. Second, convenience sampling predisposes to selection bias. Third, the overwhelming majority of participants were female (98.1%), which, while reflective of the gender distribution in the nursing workforce in China, limits the generalizability of the findings to male nurses. Fourth, recruitment from a single tertiary hospital in Wuhan limits generalizability, as the findings cannot represent the broader healthcare contexts across China. Furthermore, data collection was performed online. Variations in internet access and mobile device usage could have led to misinterpretations of some questionnaire items by participants, potentially leading to data deviations. Finally, the explanatory power of the secondary traumatic stress model was relatively low (*R* ^2^ = 0.088), which warrants further investigations with a focus on addressing those potentially omitted variables, such as trauma exposure frequency, night shift frequency, department type, and coping style.

## 6. Conclusions and Implications

This study revealed that clinical nurses from China may experience significant compassion fatigue. Among the variables included in the study, sleep quality, social support, and psychological capital are significantly associated with professional quality of life. First, it is recommended that nursing leaders educate clinical nurses about professional quality of life, raising awareness of compassion fatigue’s adverse effects while highlighting compassion satisfaction benefits. This would be operationalized through routine assessments to generate personalized reports and unit‐level analytics. Second, optimizing sleep environments, promoting healthy sleep habits, and reforming shift schedules are essential for maintaining nurses’ health, which holistically enhances professional quality of life. Furthermore, hospital administrators need to recognize the critical role of positive psychological capital in nursing. They are encouraged to evaluate and implement evidence‐based psychological capital enhancement interventions (such as mindfulness, meditation, and resilience‐based training), targeting those at high risk for compassion fatigue. Finally, it would be necessary to establish nursing policies that can foster supportive work environments and create networks for clinical nurses, as well as assist them in obtaining social support and employing proactive coping mechanisms when confronted with work stress.

## Ethics Statement

This study was approved by the Institutional Review Board (IRB) of Tongji Hospital (TJ‐IRB202404102).

## Conflicts of Interest

The authors declare no conflicts of interest.

## Author Contributions

Study design: Tian Xiong, Jing Wang, and Mei Wang. Data collection: Tian Xiong and Jing Wang. Data analysis: Tian Xiong and Mei Wang. Manuscript: Tian Xiong, Jing Wang, and Mei Wang. Tian Xiong and Jing Wang contributed equally to this work.

## Funding

No funding was received for this study.

## Data Availability

The data that support the findings of this study are available from the corresponding author upon reasonable request.
